# 12-{[4-(2-Fluoro­phen­yl)piperazin-1-yl]­meth­yl}-9α-hy­droxy-4,8-dimethyl-3,14-dioxatricyclo­[9.3.0.0^2,4^]tetra­dec-7-en-13-one

**DOI:** 10.1107/S1600536812006411

**Published:** 2012-02-17

**Authors:** Mohamed Moumou, Ahmed Benharref, Jean Claude Daran, Rachid Outouch, Moha Berraho

**Affiliations:** a Laboratoire de Chimie Bioorganique et Analytique, URAC 22, BP 146, FSTM, Université Hassan II, Mohammedia-Casablanca 20810 Mohammedia, Morocco; b Laboratoire de Chimie Biomoléculaire, Substances Naturelles et Réactivité, URAC 16, Faculté des Sciences, Semlalia, BP 2390, Boulevard My Abdellah, 40000 Marrakech, Morocco; c Laboratoire de Chimie de Coordination, 205 Route de Narbonne, 31077 Toulouse Cedex 04, France

## Abstract

The title compound, C_25_H_33_FN_2_O_4_, was synthesized from 9α-hy­droxy­parthenolide (9α-hy­droxy-4,8-dimethyl-12-methyl­ene-3,14-dioxatricyclo­[9.3.0.0^2,4^]tetra­dec-7-en-13-one), which was isolated from the chloro­form extract of the aerial parts of *Anvillea radiata*. The asymmetric unit contains two independent mol­ecules. In each mol­ecule, the ten-membered ring displays an approximative chair–chair conformation. Each of the piperazine rings adopts a perfect chair conformation, while both lactone rings show an envelope conformation, one with the C atom bearing the piperazin-1-ylmethyl group as the flap, the other with the junction C atom not attached to the ring O atom as the flap. The dihedral angles between the least-squares planes through the ten- and five-membered rings in the two mol­ecules are similar [19.1 (3) and 16.2 (3)°]. An intra­molecular O—H⋯N hydrogen bond stabilizes the mol­ecular conformation. The crystal packing is stabilized by C—H⋯O hydrogen bonds.

## Related literature

For background to the medicinal uses of the plant *Anvillea adiata*, see: El Hassany *et al.* (2004 ▶); Qureshi *et al.* (1990 ▶). For the reactivity of this sesquiterpene, see: Castaneda-Acosta *et al.* (1997 ▶); Hwang *et al.* (2006 ▶); Neukirch *et al.* (2003 ▶); Neelakantan *et al.* (2009 ▶). For ring puckering parameters, see: Cremer & Pople (1975 ▶).
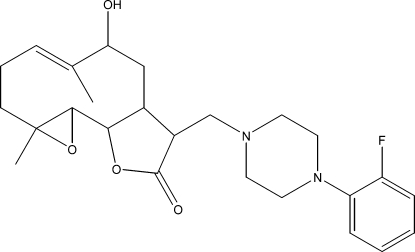


## Experimental

### 

#### Crystal data

C_25_H_33_FN_2_O_4_
*M* *_r_* = 444.53
Monoclinic, 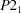

*a* = 14.583 (2) Å
*b* = 6.3366 (17) Å
*c* = 24.697 (3) Å
β = 93.598 (14)°
*V* = 2277.7 (8) Å^3^
*Z* = 4
Mo *K*α radiation
μ = 0.09 mm^−1^
*T* = 180 K
0.56 × 0.36 × 0.1 mm

#### Data collection

Agilent Xcalibur Eos Gemini ultra diffractometer
Absorption correction: multi-scan (*CrysAlis PRO*; Agilent, 2011 ▶) *T*_min_ = 0.789, *T*_max_ = 1.000
24656 measured reflections
5050 independent reflections
4582 reflections with *I* > 2σ(*I*)
*R*_int_ = 0.066

#### Refinement

*R*[*F* ^2^ > 2σ(*F* ^2^)] = 0.068
*wR*(*F* ^2^) = 0.197
*S* = 1.12
5050 reflections
583 parameters
1 restraint
H-atom parameters constrained
Δρ_max_ = 0.43 e Å^−3^
Δρ_min_ = −0.37 e Å^−3^

### 

Data collection: *CrysAlis PRO* (Agilent, 2011 ▶); cell refinement: *CrysAlis PRO*; data reduction: *CrysAlis PRO*; program(s) used to solve structure: *SHELXS97* (Sheldrick, 2008 ▶); program(s) used to refine structure: *SHELXL97* (Sheldrick, 2008 ▶); molecular graphics: *ORTEP-3 for Windows* (Farrugia, 1997 ▶) and *PLATON* (Spek, 2009 ▶); software used to prepare material for publication: *WinGX* (Farrugia, 1999 ▶).

## Supplementary Material

Crystal structure: contains datablock(s) I, global. DOI: 10.1107/S1600536812006411/bt5817sup1.cif

Structure factors: contains datablock(s) I. DOI: 10.1107/S1600536812006411/bt5817Isup2.hkl

Supplementary material file. DOI: 10.1107/S1600536812006411/bt5817Isup3.cml

Additional supplementary materials:  crystallographic information; 3D view; checkCIF report

## Figures and Tables

**Table 1:** Hydrogen-bond geometry (Å, °)

*D*—H⋯*A*	*D*—H	H⋯*A*	*D*⋯*A*	*D*—H⋯*A*
O1—H1*B*⋯N1	0.82	2.19	2.979 (6)	163
O1*A*—H1*A*1⋯N2*A*	0.82	2.08	2.882 (6)	164
C1—H1⋯O3^i^	0.98	2.47	3.035 (7)	116
C13—H131⋯O3^ii^	0.97	2.51	3.394 (7)	152
C10—H10⋯O1	0.98	2.36	2.861 (6)	111
C10*A*—H10*A*⋯O1*A*	0.98	2.36	2.860 (6)	111
C13*A*—H13*A*⋯O3*A*^iii^	0.97	2.57	3.391 (6)	143
C11*A*—H11*A*⋯O3*A*^iv^	0.98	2.60	3.338 (6)	132
C15—H15*F*⋯O1^v^	0.96	2.42	3.375 (7)	171

Symmetry codes: (i) 
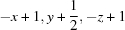
; (ii) 
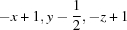
; (iii) 
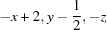
; (iv) 
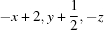
; (v) 
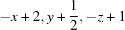
.

**Fig. 1.**
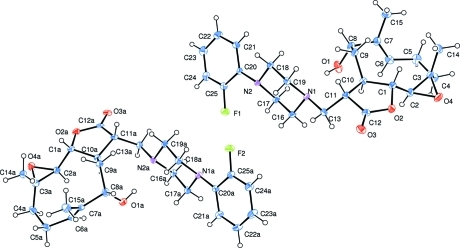
: Molecular structure of the title compound with displacement ellipsoids drawn at the 30% probability level. H atoms are represented as small spheres of arbitrary radii.

**Fig. 2.**
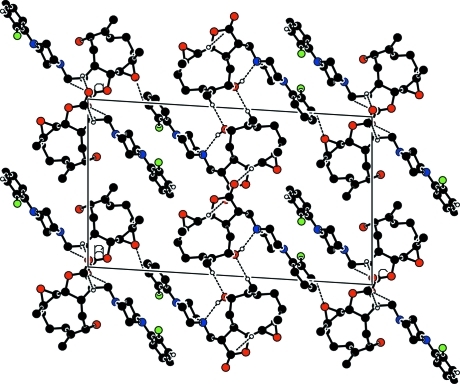
: Partial packing view showing the C–H···O hydrogen bonds as dashed lines. H atoms not involved in hydrogen bonding have been omitted for clarity.

## supplementary crystallographic information

### Comment

Our work lies within the framework of the evaluation of medicinal plants and in particular, *Anvillea radiata*. The main constituent of the chloroform extract of aerial parts of this plant is 9α-hydroxypartenolide(El Hassany *et al.*, 2004). The reactivity of this sesquiterpene lactone and its derivatives has been the subject of several studies(Castaneda-Acosta *et al.*,1997; Neukirch *et al.*, 2003; Hwang *et al.*, 2006 Neelakantan *et al.*, 2009), in order to prepare products with high value which can be used in the pharmacological industry. In this context,we have treated the 9α-hydroxyparthenolide with an equivalent amount of 1-(2- fluorophenyl)piperazine and isolated the 9α-Hydroxy-4,8- dimethyl-12-[(4-(2-fluorophenyl)piperazin-1-yl)methyl)]- 3,14-dioxatricyclo-[9.3.0.0^2^,^4^]tetradec-7-en-13-one with a yield of 95%. The structure of this new product was confirmed by its single-crystal X-ray structure. The asymmetric unit contains two crystallographically independent molecules(Fig.1). Each molecule is built up from two fused five-and ten-membered rings with the fluorophenylpiperazine group as a substituent. The ten-membered ring displays an approximate chair-chair conformation. Whereas the lactone ring (O2A, C1A···C12A) adopt an envelope conformation, as indicated by the puckering parameters Q = 0.229 (5) Å and φ = 78.9 (11)°(Cremer & Pople, 1975), the other lactone ring (O2,C1···C12)shows a twisted conformation with Q = 0.204 (5) Å and φ =47.9 (14)°. The piperazine ring, in the two molecules, has a perfect chair conformation with QT = 0.594 (5) Å, θ = 176.5 (5)° and φ2 =9.0 (9)° for the ring (N1A,C17A···C18A)and QT = 0.587 (5) Å, θ = 176.2 (5)° andφ =187.0 (7)° for the other piperazine ring (N1,C16···C19).In the first molecule (C1 to C25), the dihedral angle between the mean planes of the ten-membered ring and the lactone ring is 16.2 (3).The corresponding value in the second molecule (C1A to C25A) is 19.3 (3) °. In the crystal, C—H···O hydrogen bonding links the molecules into sheets lying parallel to the *c* axis (Table 1, Fig.2). In addition, the molecular conformation is stabilized by an O—H···N hydrogen bond between the hydroxy group and a piperazine N atom.

### Experimental

The mixture of 9α-hydroxyparthenolide (0.5 g, 2 mmol) and one equivalent of 1-(2- fluorophenyl) piperazine in EtOH (20 ml) was stirred for one night at room temperature. The next day, the reaction was stopped by adding water (10 ml) and extracted three times with ethyl acetate (3 *x* 20 ml). The combined organic layers were dried over anhydrous MgSO_4_, filtered and concentrated under vacuum to give 1 g (1.9 mmol)of the title compound (yield: 95%) which was recrystallized in ethyle acetate.

### Refinement

All H atoms were fixed geometrically and treated as riding with O—H = 0.82 Å, C—H = 0.96 Å (methyl), 0.97 Å (methylene), 0.98 Å (methine) with *U*_iso_(H) = 1.2*U*_eq_(methylene, methine) or *U*_iso_(H) = 1.5*U*_eq_(methyl, OH). In the absence of significant anomalous scattering, the absolute configuration could not be reliably determined and thus Friedel pairs were merged and any references to the Flack parameter were removed.

### Crystal data

**Table:**

C_25_H_33_FN_2_O_4_	*F*(000) = 952
*M**_r_* = 444.53	*D*_x_ = 1.296 Mg m^−^^3^
Monoclinic, *P*2_1_	Mo *K*α radiation, λ = 0.71073 Å
Hall symbol: P 2yb	Cell parameters from 24632 reflections
*a* = 14.583 (2) Å	θ = 3.5–26.4°
*b* = 6.3366 (17) Å	µ = 0.09 mm^−^^1^
*c* = 24.697 (3) Å	*T* = 180 K
β = 93.598 (14)°	Block, colourless
*V* = 2277.7 (8) Å^3^	0.56 × 0.36 × 0.1 mm
*Z* = 4	

### Data collection

**Table:**

Agilent Xcalibur Eos Gemini ultra diffractometer	5052 independent reflections
Radiation source: fine-focus sealed tube	4582 reflections with *I* > 2σ(*I*)
Graphite monochromator	*R*_int_ = 0.066
Detector resolution: 16.1978 pixels mm^-1^	θ_max_ = 26.4°, θ_min_ = 3.5°
ω scans	*h* = −18→18
Absorption correction: multi-scan (*CrysAlis PRO*; Agilent, 2011)	*k* = −7→7
*T*_min_ = 0.789, *T*_max_ = 1.000	*l* = −30→30
24656 measured reflections	

### Refinement

**Table:**

Refinement on *F*^2^	Primary atom site location: structure-invariant direct methods
Least-squares matrix: full	Secondary atom site location: difference Fourier map
*R*[*F*^2^ > 2σ(*F*^2^)] = 0.068	Hydrogen site location: inferred from neighbouring sites
*wR*(*F*^2^) = 0.197	H-atom parameters constrained
*S* = 1.12	*w* = 1/[σ^2^(*F*_o_^2^) + (0.0994*P*)^2^ + 2.617*P*] where *P* = (*F*_o_^2^ + 2*F*_c_^2^)/3
5050 reflections	(Δ/σ)_max_ = 0.002
583 parameters	Δρ_max_ = 0.43 e Å^−^^3^
1 restraint	Δρ_min_ = −0.37 e Å^−^^3^

### Special details

**Table:**

Experimental. Empirical absorption correction using spherical harmonics, implemented in SCALE3 ABSPACK scaling algorithm. CrysAlisPro (Agilent Technologies,2011)
Geometry. All s.u.'s (except the s.u. in the dihedral angle between two l.s. planes) are estimated using the full covariance matrix. The cell s.u.'s are taken into account individually in the estimation of s.u.'s in distances, angles and torsion angles; correlations between s.u.'s in cell parameters are only used when they are defined by crystal symmetry. An approximate (isotropic) treatment of cell s.u.'s is used for estimating s.u.'s involving l.s. planes.
Refinement. Refinement of *F*^2^ against ALL reflections. The weighted *R*-factor *wR* and goodness of fit *S* are based on *F*^2^, conventional *R*-factors *R* are based on *F*, with *F* set to zero for negative *F*^2^. The threshold expression of *F*^2^ > 2σ(*F*^2^) is used only for calculating *R*-factors(gt) *etc*. and is not relevant to the choice of reflections for refinement. *R*-factors based on *F*^2^ are statistically about twice as large as those based on *F*, and *R*- factors based on ALL data will be even larger.

### Fractional atomic coordinates and isotropic or equivalent isotropic displacement parameters (Å^2^)

**Table:**

	*x*	*y*	*z*	*U*_iso_*/*U*_eq_	
C1A	0.8424 (3)	−0.1376 (8)	−0.07964 (18)	0.0227 (10)	
H1A	0.8190	−0.0141	−0.1000	0.027*	
C2A	0.8236 (3)	−0.3358 (8)	−0.11130 (18)	0.0250 (10)	
H2A	0.8336	−0.4649	−0.0899	0.030*	
C3A	0.7535 (3)	−0.3580 (9)	−0.15656 (19)	0.0270 (11)	
C4A	0.7080 (4)	−0.5696 (10)	−0.1628 (2)	0.0353 (13)	
H4A1	0.7490	−0.6773	−0.1473	0.042*	
H4A2	0.6962	−0.6005	−0.2011	0.042*	
C5A	0.6168 (4)	−0.5769 (10)	−0.1346 (2)	0.0369 (13)	
H5A1	0.5703	−0.4981	−0.1559	0.044*	
H5A2	0.5962	−0.7220	−0.1324	0.044*	
C6A	0.6282 (4)	−0.4847 (9)	−0.0784 (2)	0.0314 (12)	
H6A	0.6657	−0.5600	−0.0535	0.038*	
C7A	0.5916 (3)	−0.3105 (10)	−0.0603 (2)	0.0301 (12)	
C8A	0.6277 (3)	−0.2092 (10)	−0.00794 (19)	0.0313 (12)	
H8A	0.5774	−0.1310	0.0073	0.038*	
C9A	0.7044 (3)	−0.0507 (8)	−0.01998 (19)	0.0232 (10)	
H9A1	0.6876	0.0201	−0.0540	0.028*	
H9A2	0.7080	0.0557	0.0083	0.028*	
C10A	0.8004 (3)	−0.1502 (8)	−0.02369 (17)	0.0197 (9)	
H10A	0.7951	−0.3001	−0.0146	0.024*	
C11A	0.8758 (3)	−0.0569 (8)	0.01478 (18)	0.0212 (9)	
H11A	0.8639	0.0942	0.0191	0.025*	
C12A	0.9618 (3)	−0.0835 (7)	−0.01515 (19)	0.0210 (10)	
C13A	0.8871 (3)	−0.1570 (9)	0.07060 (17)	0.0250 (10)	
H13A	0.9092	−0.3004	0.0671	0.030*	
H13B	0.9330	−0.0790	0.0926	0.030*	
C14A	0.7011 (4)	−0.1738 (9)	−0.1810 (2)	0.0303 (11)	
H14A	0.7399	−0.0512	−0.1798	0.045*	
H14B	0.6482	−0.1471	−0.1608	0.045*	
H14C	0.6818	−0.2052	−0.2180	0.045*	
C15A	0.5173 (4)	−0.1808 (11)	−0.0897 (2)	0.0382 (13)	
H15A	0.5068	−0.2325	−0.1261	0.057*	
H15B	0.5363	−0.0358	−0.0907	0.057*	
H15C	0.4617	−0.1915	−0.0711	0.057*	
C16A	0.8080 (3)	−0.3108 (8)	0.14370 (18)	0.0235 (10)	
H16A	0.8558	−0.2650	0.1702	0.028*	
H16B	0.8249	−0.4487	0.1305	0.028*	
C17A	0.7173 (3)	−0.3263 (8)	0.17053 (18)	0.0226 (10)	
H17A	0.6700	−0.3781	0.1445	0.027*	
H17B	0.7232	−0.4254	0.2005	0.027*	
C18A	0.6868 (3)	0.0355 (8)	0.14613 (19)	0.0244 (10)	
H18A	0.6717	0.1734	0.1601	0.029*	
H18B	0.6389	−0.0045	0.1191	0.029*	
C19A	0.7782 (3)	0.0465 (8)	0.12006 (19)	0.0240 (10)	
H19A	0.7747	0.1502	0.0911	0.029*	
H19B	0.8260	0.0901	0.1468	0.029*	
C20A	0.6142 (3)	−0.1175 (8)	0.22233 (18)	0.0204 (10)	
C21A	0.5586 (3)	−0.2906 (9)	0.22979 (19)	0.0263 (11)	
H21A	0.5701	−0.4161	0.2119	0.032*	
C22A	0.4857 (3)	−0.2809 (9)	0.2634 (2)	0.0325 (12)	
H22A	0.4501	−0.4005	0.2681	0.039*	
C23A	0.4654 (4)	−0.0980 (10)	0.2898 (2)	0.0358 (13)	
H23A	0.4156	−0.0920	0.3115	0.043*	
C24A	0.5203 (4)	0.0784 (9)	0.2835 (2)	0.0320 (12)	
H24A	0.5080	0.2040	0.3011	0.038*	
C25A	0.5934 (3)	0.0646 (8)	0.25078 (19)	0.0251 (10)	
O1A	0.6593 (3)	−0.3633 (8)	0.03028 (14)	0.0375 (10)	
H1A1	0.6922	−0.3071	0.0543	0.056*	
O2A	0.9417 (2)	−0.1221 (6)	−0.06803 (13)	0.0256 (7)	
O3A	1.0412 (2)	−0.0722 (6)	0.00239 (14)	0.0281 (8)	
O4A	0.8513 (2)	−0.3533 (7)	−0.16622 (13)	0.0319 (8)	
N1A	0.6909 (3)	−0.1189 (7)	0.19052 (15)	0.0215 (8)	
N2A	0.8010 (3)	−0.1602 (7)	0.09816 (14)	0.0218 (8)	
F2	0.6494 (2)	0.2337 (5)	0.24852 (12)	0.0347 (7)	
C1	0.6454 (3)	0.8975 (9)	0.56683 (18)	0.0250 (10)	
H1	0.6460	1.0484	0.5755	0.030*	
C2	0.6873 (3)	0.7724 (9)	0.61320 (18)	0.0291 (11)	
H2	0.7046	0.6286	0.6034	0.035*	
C3	0.7433 (3)	0.8598 (10)	0.65909 (19)	0.0272 (11)	
C4	0.8206 (4)	0.7248 (10)	0.6835 (2)	0.0347 (13)	
H4A	0.8043	0.5771	0.6792	0.042*	
H4B	0.8293	0.7541	0.7220	0.042*	
C5	0.9112 (4)	0.7675 (11)	0.6563 (2)	0.0369 (13)	
H5A	0.9366	0.9009	0.6693	0.044*	
H5B	0.9551	0.6574	0.6666	0.044*	
C6	0.8975 (3)	0.7746 (9)	0.5959 (2)	0.0312 (12)	
H6	0.8841	0.6467	0.5787	0.037*	
C7	0.9021 (3)	0.9422 (10)	0.5638 (2)	0.0296 (11)	
C8	0.8663 (3)	0.9337 (10)	0.5053 (2)	0.0306 (12)	
H8	0.9049	1.0253	0.4843	0.037*	
C9	0.7670 (3)	1.0168 (9)	0.49891 (19)	0.0269 (11)	
H9A	0.7541	1.0603	0.4616	0.032*	
H9B	0.7622	1.1408	0.5216	0.032*	
C10	0.6936 (3)	0.8562 (8)	0.51388 (17)	0.0212 (9)	
H10	0.7232	0.7176	0.5172	0.025*	
C11	0.6141 (3)	0.8380 (8)	0.47070 (18)	0.0220 (10)	
H11	0.6087	0.9701	0.4501	0.026*	
C12	0.5300 (3)	0.8096 (8)	0.50258 (19)	0.0231 (10)	
C13	0.6249 (3)	0.6545 (9)	0.43165 (18)	0.0254 (10)	
H131	0.6225	0.5228	0.4515	0.030*	
H132	0.5739	0.6556	0.4045	0.030*	
C14	0.7548 (4)	1.0945 (10)	0.6683 (2)	0.0343 (13)	
H14D	0.7033	1.1678	0.6511	0.051*	
H14E	0.8103	1.1413	0.6533	0.051*	
H14F	0.7579	1.1232	0.7066	0.051*	
C15	0.9342 (4)	1.1565 (11)	0.5808 (2)	0.0395 (14)	
H15D	0.9490	1.1575	0.6192	0.059*	
H15E	0.8864	1.2573	0.5721	0.059*	
H15F	0.9877	1.1928	0.5621	0.059*	
C16	0.7315 (4)	0.4575 (8)	0.3813 (2)	0.0272 (11)	
H16C	0.6828	0.4194	0.3545	0.033*	
H16D	0.7337	0.3517	0.4097	0.033*	
C17	0.8227 (3)	0.4615 (8)	0.35471 (19)	0.0261 (11)	
H17C	0.8719	0.4938	0.3817	0.031*	
H17D	0.8347	0.3238	0.3395	0.031*	
C18	0.7965 (3)	0.8276 (8)	0.33228 (18)	0.0219 (10)	
H18C	0.7911	0.9279	0.3026	0.026*	
H18D	0.8448	0.8760	0.3581	0.026*	
C19	0.7061 (3)	0.8165 (8)	0.35974 (18)	0.0229 (10)	
H19C	0.6913	0.9548	0.3736	0.027*	
H19D	0.6573	0.7753	0.3334	0.027*	
C20	0.8968 (3)	0.6153 (8)	0.27872 (17)	0.0192 (9)	
C21	0.9531 (3)	0.7883 (9)	0.27203 (18)	0.0250 (10)	
H21	0.9426	0.9125	0.2907	0.030*	
C22	1.0259 (3)	0.7785 (11)	0.2375 (2)	0.0321 (12)	
H22	1.0636	0.8953	0.2337	0.039*	
C23	1.0416 (3)	0.5970 (11)	0.2094 (2)	0.0343 (13)	
H23	1.0898	0.5908	0.1866	0.041*	
C24	0.9860 (3)	0.4236 (10)	0.2149 (2)	0.0289 (11)	
H24	0.9969	0.2990	0.1965	0.035*	
C25	0.9135 (3)	0.4382 (8)	0.24841 (19)	0.0226 (10)	
O1	0.8713 (3)	0.7258 (8)	0.48416 (15)	0.0429 (11)	
H1B	0.8355	0.7144	0.4574	0.064*	
O2	0.5509 (2)	0.8223 (6)	0.55579 (13)	0.0275 (8)	
O3	0.4522 (2)	0.7769 (7)	0.48496 (15)	0.0331 (9)	
O4	0.6512 (3)	0.7882 (8)	0.66572 (13)	0.0384 (10)	
N1	0.7116 (3)	0.6642 (7)	0.40444 (14)	0.0206 (8)	
N2	0.8201 (3)	0.6209 (7)	0.31174 (15)	0.0197 (8)	
F1	0.8570 (2)	0.2681 (5)	0.25078 (12)	0.0337 (7)	

### Atomic displacement parameters (Å^2^)

**Table:**

	*U*^11^	*U*^22^	*U*^33^	*U*^12^	*U*^13^	*U*^23^
C1A	0.021 (2)	0.021 (2)	0.027 (2)	−0.0018 (19)	0.0038 (17)	0.0016 (19)
C2A	0.031 (2)	0.020 (2)	0.024 (2)	−0.001 (2)	0.0039 (18)	0.000 (2)
C3A	0.031 (3)	0.025 (3)	0.025 (2)	−0.001 (2)	0.0054 (19)	−0.003 (2)
C4A	0.045 (3)	0.030 (3)	0.031 (3)	−0.001 (3)	−0.005 (2)	−0.007 (2)
C5A	0.044 (3)	0.027 (3)	0.038 (3)	−0.008 (3)	−0.005 (2)	−0.001 (2)
C6A	0.028 (3)	0.031 (3)	0.035 (3)	−0.012 (2)	−0.001 (2)	0.007 (2)
C7A	0.023 (2)	0.038 (3)	0.030 (2)	−0.017 (2)	0.0050 (18)	0.002 (2)
C8A	0.026 (2)	0.043 (3)	0.025 (2)	−0.005 (3)	0.0047 (18)	−0.001 (2)
C9A	0.019 (2)	0.026 (3)	0.025 (2)	0.000 (2)	0.0019 (16)	0.000 (2)
C10A	0.020 (2)	0.019 (2)	0.021 (2)	−0.0030 (19)	0.0028 (16)	0.0021 (18)
C11A	0.017 (2)	0.019 (2)	0.028 (2)	−0.0022 (19)	0.0030 (17)	0.0000 (19)
C12A	0.019 (2)	0.012 (2)	0.033 (2)	−0.0045 (18)	0.0051 (18)	0.0004 (19)
C13A	0.026 (2)	0.029 (3)	0.020 (2)	0.001 (2)	0.0014 (17)	−0.001 (2)
C14A	0.038 (3)	0.028 (3)	0.025 (2)	−0.004 (2)	−0.0017 (19)	0.002 (2)
C15A	0.030 (3)	0.043 (3)	0.041 (3)	−0.001 (3)	−0.006 (2)	−0.002 (3)
C16A	0.026 (2)	0.021 (2)	0.023 (2)	0.007 (2)	0.0014 (17)	0.0021 (19)
C17A	0.029 (2)	0.017 (2)	0.022 (2)	0.005 (2)	0.0024 (17)	0.0029 (19)
C18A	0.029 (2)	0.019 (2)	0.026 (2)	0.007 (2)	0.0045 (18)	0.004 (2)
C19A	0.034 (3)	0.019 (2)	0.019 (2)	0.001 (2)	0.0044 (18)	−0.0010 (18)
C20A	0.018 (2)	0.019 (2)	0.024 (2)	0.0019 (19)	−0.0004 (16)	0.0030 (19)
C21A	0.023 (2)	0.025 (3)	0.030 (2)	0.005 (2)	0.0014 (18)	0.004 (2)
C22A	0.021 (2)	0.030 (3)	0.048 (3)	−0.004 (2)	0.009 (2)	0.007 (3)
C23A	0.022 (2)	0.037 (3)	0.050 (3)	0.004 (2)	0.017 (2)	0.006 (3)
C24A	0.034 (3)	0.026 (3)	0.038 (3)	0.005 (2)	0.012 (2)	0.001 (2)
C25A	0.025 (2)	0.023 (3)	0.027 (2)	0.002 (2)	0.0029 (18)	0.004 (2)
O1A	0.041 (2)	0.046 (2)	0.0252 (18)	−0.021 (2)	0.0003 (15)	0.0071 (18)
O2A	0.0223 (17)	0.0273 (19)	0.0280 (16)	−0.0037 (15)	0.0084 (12)	0.0013 (14)
O3A	0.0203 (17)	0.0241 (19)	0.0400 (19)	−0.0054 (15)	0.0029 (14)	−0.0019 (16)
O4A	0.0317 (18)	0.039 (2)	0.0257 (17)	−0.0007 (18)	0.0085 (13)	−0.0078 (17)
N1A	0.024 (2)	0.017 (2)	0.0239 (19)	0.0023 (16)	0.0051 (15)	0.0017 (16)
N2A	0.030 (2)	0.018 (2)	0.0174 (17)	0.0042 (18)	0.0037 (14)	−0.0008 (16)
F2	0.0390 (17)	0.0206 (16)	0.0460 (17)	−0.0065 (14)	0.0151 (13)	−0.0069 (14)
C1	0.028 (2)	0.024 (2)	0.024 (2)	0.000 (2)	0.0035 (18)	0.0011 (19)
C2	0.037 (3)	0.029 (3)	0.022 (2)	−0.002 (2)	0.0046 (19)	0.003 (2)
C3	0.024 (2)	0.035 (3)	0.023 (2)	−0.005 (2)	0.0048 (18)	0.002 (2)
C4	0.042 (3)	0.035 (3)	0.026 (2)	−0.001 (3)	−0.004 (2)	0.008 (2)
C5	0.030 (3)	0.042 (3)	0.038 (3)	0.012 (3)	−0.005 (2)	0.006 (3)
C6	0.024 (2)	0.029 (3)	0.040 (3)	0.010 (2)	0.000 (2)	−0.007 (2)
C7	0.020 (2)	0.039 (3)	0.030 (2)	−0.002 (2)	0.0042 (18)	−0.001 (2)
C8	0.021 (2)	0.042 (3)	0.029 (3)	−0.003 (2)	0.0067 (19)	0.000 (2)
C9	0.025 (2)	0.033 (3)	0.022 (2)	−0.005 (2)	0.0001 (17)	0.003 (2)
C10	0.020 (2)	0.020 (2)	0.024 (2)	0.0021 (19)	0.0029 (16)	0.0028 (19)
C11	0.018 (2)	0.023 (2)	0.025 (2)	0.000 (2)	0.0033 (17)	−0.0005 (19)
C12	0.016 (2)	0.020 (3)	0.034 (2)	0.0014 (19)	0.0056 (18)	−0.004 (2)
C13	0.021 (2)	0.029 (3)	0.026 (2)	−0.004 (2)	0.0085 (17)	−0.004 (2)
C14	0.035 (3)	0.035 (3)	0.033 (3)	−0.001 (3)	0.004 (2)	−0.009 (2)
C15	0.032 (3)	0.047 (4)	0.039 (3)	−0.018 (3)	0.000 (2)	0.002 (3)
C16	0.036 (3)	0.019 (2)	0.028 (2)	−0.003 (2)	0.012 (2)	0.001 (2)
C17	0.035 (3)	0.019 (2)	0.025 (2)	0.005 (2)	0.0078 (19)	0.003 (2)
C18	0.024 (2)	0.019 (2)	0.023 (2)	0.003 (2)	0.0054 (17)	−0.0001 (19)
C19	0.025 (2)	0.021 (2)	0.024 (2)	0.006 (2)	0.0039 (17)	0.001 (2)
C20	0.018 (2)	0.024 (2)	0.016 (2)	0.002 (2)	0.0022 (15)	0.0005 (19)
C21	0.020 (2)	0.028 (3)	0.027 (2)	−0.004 (2)	0.0022 (17)	−0.001 (2)
C22	0.020 (2)	0.040 (3)	0.037 (3)	−0.004 (2)	0.0053 (19)	0.005 (3)
C23	0.019 (2)	0.050 (4)	0.035 (3)	0.005 (3)	0.0083 (19)	0.002 (3)
C24	0.028 (2)	0.032 (3)	0.027 (2)	0.013 (2)	0.0036 (18)	0.000 (2)
C25	0.019 (2)	0.022 (2)	0.026 (2)	0.004 (2)	0.0001 (17)	0.001 (2)
O1	0.032 (2)	0.060 (3)	0.036 (2)	0.014 (2)	0.0034 (16)	−0.018 (2)
O2	0.0257 (17)	0.033 (2)	0.0243 (16)	−0.0030 (16)	0.0072 (12)	−0.0023 (15)
O3	0.0195 (17)	0.040 (2)	0.040 (2)	−0.0038 (17)	0.0028 (14)	−0.0114 (18)
O4	0.0328 (19)	0.055 (3)	0.0280 (17)	−0.018 (2)	0.0059 (14)	0.0071 (19)
N1	0.0229 (19)	0.020 (2)	0.0190 (18)	−0.0011 (17)	0.0058 (14)	0.0015 (16)
N2	0.0219 (19)	0.0167 (19)	0.0212 (18)	0.0000 (17)	0.0076 (14)	−0.0002 (16)
F1	0.0417 (17)	0.0220 (16)	0.0382 (16)	−0.0036 (15)	0.0087 (13)	−0.0061 (13)

### Geometric parameters (Å, º)

**Table:**

C1A—O2A	1.462 (5)	C1—O2	1.468 (6)
C1A—C2A	1.496 (7)	C1—C2	1.492 (7)
C1A—C10A	1.548 (6)	C1—C10	1.545 (6)
C1A—H1A	0.9800	C1—H1	0.9800
C2A—O4A	1.444 (5)	C2—O4	1.434 (6)
C2A—C3A	1.473 (7)	C2—C3	1.464 (7)
C2A—H2A	0.9800	C2—H2	0.9800
C3A—O4A	1.461 (6)	C3—O4	1.437 (6)
C3A—C4A	1.500 (8)	C3—C4	1.510 (7)
C3A—C14A	1.501 (8)	C3—C14	1.512 (8)
C4A—C5A	1.540 (8)	C4—C5	1.543 (8)
C4A—H4A1	0.9700	C4—H4A	0.9700
C4A—H4A2	0.9700	C4—H4B	0.9700
C5A—C6A	1.504 (7)	C5—C6	1.494 (7)
C5A—H5A1	0.9700	C5—H5A	0.9700
C5A—H5A2	0.9700	C5—H5B	0.9700
C6A—C7A	1.317 (8)	C6—C7	1.330 (8)
C6A—H6A	0.9300	C6—H6	0.9300
C7A—C8A	1.508 (7)	C7—C15	1.488 (9)
C7A—C15A	1.509 (8)	C7—C8	1.506 (7)
C8A—O1A	1.415 (7)	C8—O1	1.420 (8)
C8A—C9A	1.546 (7)	C8—C9	1.540 (7)
C8A—H8A	0.9800	C8—H8	0.9800
C9A—C10A	1.543 (6)	C9—C10	1.540 (7)
C9A—H9A1	0.9700	C9—H9A	0.9700
C9A—H9A2	0.9700	C9—H9B	0.9700
C10A—C11A	1.526 (6)	C10—C11	1.529 (6)
C10A—H10A	0.9800	C10—H10	0.9800
C11A—C12A	1.506 (6)	C11—C12	1.510 (6)
C11A—C13A	1.517 (6)	C11—C13	1.525 (7)
C11A—H11A	0.9800	C11—H11	0.9800
C12A—O3A	1.212 (5)	C12—O3	1.207 (5)
C12A—O2A	1.343 (6)	C12—O2	1.333 (6)
C13A—N2A	1.466 (6)	C13—N1	1.470 (5)
C13A—H13A	0.9700	C13—H131	0.9700
C13A—H13B	0.9700	C13—H132	0.9700
C14A—H14A	0.9600	C14—H14D	0.9600
C14A—H14B	0.9600	C14—H14E	0.9600
C14A—H14C	0.9600	C14—H14F	0.9600
C15A—H15A	0.9600	C15—H15D	0.9600
C15A—H15B	0.9600	C15—H15E	0.9600
C15A—H15C	0.9600	C15—H15F	0.9600
C16A—N2A	1.474 (6)	C16—N1	1.465 (7)
C16A—C17A	1.520 (6)	C16—C17	1.520 (6)
C16A—H16A	0.9700	C16—H16C	0.9700
C16A—H16B	0.9700	C16—H16D	0.9700
C17A—N1A	1.464 (6)	C17—N2	1.464 (6)
C17A—H17A	0.9700	C17—H17C	0.9700
C17A—H17B	0.9700	C17—H17D	0.9700
C18A—N1A	1.468 (6)	C18—N2	1.454 (6)
C18A—C19A	1.518 (6)	C18—C19	1.522 (6)
C18A—H18A	0.9700	C18—H18C	0.9700
C18A—H18B	0.9700	C18—H18D	0.9700
C19A—N2A	1.463 (6)	C19—N1	1.465 (6)
C19A—H19A	0.9700	C19—H19C	0.9700
C19A—H19B	0.9700	C19—H19D	0.9700
C20A—C21A	1.384 (7)	C20—C25	1.379 (7)
C20A—C25A	1.394 (7)	C20—C21	1.386 (7)
C20A—N1A	1.407 (6)	C20—N2	1.426 (5)
C21A—C22A	1.391 (7)	C21—C22	1.404 (6)
C21A—H21A	0.9300	C21—H21	0.9300
C22A—C23A	1.370 (9)	C22—C23	1.370 (9)
C22A—H22A	0.9300	C22—H22	0.9300
C23A—C24A	1.389 (8)	C23—C24	1.377 (9)
C23A—H23A	0.9300	C23—H23	0.9300
C24A—C25A	1.380 (7)	C24—C25	1.385 (6)
C24A—H24A	0.9300	C24—H24	0.9300
C25A—F2	1.351 (6)	C25—F1	1.361 (6)
O1A—H1A1	0.8200	O1—H1B	0.8200
			
O2A—C1A—C2A	107.8 (4)	O2—C1—C2	107.7 (4)
O2A—C1A—C10A	105.7 (3)	O2—C1—C10	105.0 (4)
C2A—C1A—C10A	110.7 (4)	C2—C1—C10	112.0 (4)
O2A—C1A—H1A	110.8	O2—C1—H1	110.6
C2A—C1A—H1A	110.8	C2—C1—H1	110.6
C10A—C1A—H1A	110.8	C10—C1—H1	110.6
O4A—C2A—C3A	60.1 (3)	O4—C2—C3	59.5 (3)
O4A—C2A—C1A	120.2 (4)	O4—C2—C1	120.1 (5)
C3A—C2A—C1A	124.9 (4)	C3—C2—C1	125.1 (5)
O4A—C2A—H2A	113.7	O4—C2—H2	113.8
C3A—C2A—H2A	113.7	C3—C2—H2	113.8
C1A—C2A—H2A	113.7	C1—C2—H2	113.8
O4A—C3A—C2A	58.9 (3)	O4—C3—C2	59.2 (3)
O4A—C3A—C4A	115.6 (5)	O4—C3—C4	117.1 (5)
C2A—C3A—C4A	116.4 (5)	C2—C3—C4	117.5 (5)
O4A—C3A—C14A	113.3 (4)	O4—C3—C14	112.9 (5)
C2A—C3A—C14A	122.8 (5)	C2—C3—C14	122.7 (5)
C4A—C3A—C14A	116.3 (4)	C4—C3—C14	115.1 (5)
C3A—C4A—C5A	111.7 (5)	C3—C4—C5	111.4 (4)
C3A—C4A—H4A1	109.3	C3—C4—H4A	109.4
C5A—C4A—H4A1	109.3	C5—C4—H4A	109.4
C3A—C4A—H4A2	109.3	C3—C4—H4B	109.4
C5A—C4A—H4A2	109.3	C5—C4—H4B	109.4
H4A1—C4A—H4A2	107.9	H4A—C4—H4B	108.0
C6A—C5A—C4A	110.9 (4)	C6—C5—C4	112.1 (4)
C6A—C5A—H5A1	109.5	C6—C5—H5A	109.2
C4A—C5A—H5A1	109.5	C4—C5—H5A	109.2
C6A—C5A—H5A2	109.5	C6—C5—H5B	109.2
C4A—C5A—H5A2	109.5	C4—C5—H5B	109.2
H5A1—C5A—H5A2	108.0	H5A—C5—H5B	107.9
C7A—C6A—C5A	128.0 (5)	C7—C6—C5	127.7 (6)
C7A—C6A—H6A	116.0	C7—C6—H6	116.2
C5A—C6A—H6A	116.0	C5—C6—H6	116.2
C6A—C7A—C8A	121.4 (5)	C6—C7—C15	125.9 (5)
C6A—C7A—C15A	125.9 (5)	C6—C7—C8	121.0 (5)
C8A—C7A—C15A	112.5 (5)	C15—C7—C8	112.8 (5)
O1A—C8A—C7A	111.1 (5)	O1—C8—C7	111.3 (5)
O1A—C8A—C9A	111.6 (4)	O1—C8—C9	110.4 (4)
C7A—C8A—C9A	109.3 (4)	C7—C8—C9	110.7 (4)
O1A—C8A—H8A	108.3	O1—C8—H8	108.1
C7A—C8A—H8A	108.3	C7—C8—H8	108.1
C9A—C8A—H8A	108.3	C9—C8—H8	108.1
C10A—C9A—C8A	114.5 (4)	C8—C9—C10	114.4 (5)
C10A—C9A—H9A1	108.6	C8—C9—H9A	108.7
C8A—C9A—H9A1	108.6	C10—C9—H9A	108.7
C10A—C9A—H9A2	108.6	C8—C9—H9B	108.7
C8A—C9A—H9A2	108.6	C10—C9—H9B	108.7
H9A1—C9A—H9A2	107.6	H9A—C9—H9B	107.6
C11A—C10A—C9A	115.2 (4)	C11—C10—C9	113.0 (4)
C11A—C10A—C1A	103.1 (3)	C11—C10—C1	103.8 (4)
C9A—C10A—C1A	116.3 (4)	C9—C10—C1	116.8 (4)
C11A—C10A—H10A	107.3	C11—C10—H10	107.6
C9A—C10A—H10A	107.3	C9—C10—H10	107.6
C1A—C10A—H10A	107.3	C1—C10—H10	107.6
C12A—C11A—C13A	110.7 (4)	C12—C11—C13	111.0 (4)
C12A—C11A—C10A	103.9 (4)	C12—C11—C10	104.5 (3)
C13A—C11A—C10A	115.9 (4)	C13—C11—C10	113.3 (4)
C12A—C11A—H11A	108.7	C12—C11—H11	109.3
C13A—C11A—H11A	108.7	C13—C11—H11	109.3
C10A—C11A—H11A	108.7	C10—C11—H11	109.3
O3A—C12A—O2A	120.3 (4)	O3—C12—O2	121.2 (4)
O3A—C12A—C11A	128.6 (4)	O3—C12—C11	127.5 (4)
O2A—C12A—C11A	111.1 (4)	O2—C12—C11	111.3 (4)
N2A—C13A—C11A	112.3 (4)	N1—C13—C11	112.5 (4)
N2A—C13A—H13A	109.1	N1—C13—H131	109.1
C11A—C13A—H13A	109.1	C11—C13—H131	109.1
N2A—C13A—H13B	109.1	N1—C13—H132	109.1
C11A—C13A—H13B	109.1	C11—C13—H132	109.1
H13A—C13A—H13B	107.9	H131—C13—H132	107.8
C3A—C14A—H14A	109.5	C3—C14—H14D	109.5
C3A—C14A—H14B	109.5	C3—C14—H14E	109.5
H14A—C14A—H14B	109.5	H14D—C14—H14E	109.5
C3A—C14A—H14C	109.5	C3—C14—H14F	109.5
H14A—C14A—H14C	109.5	H14D—C14—H14F	109.5
H14B—C14A—H14C	109.5	H14E—C14—H14F	109.5
C7A—C15A—H15A	109.5	C7—C15—H15D	109.5
C7A—C15A—H15B	109.5	C7—C15—H15E	109.5
H15A—C15A—H15B	109.5	H15D—C15—H15E	109.5
C7A—C15A—H15C	109.5	C7—C15—H15F	109.5
H15A—C15A—H15C	109.5	H15D—C15—H15F	109.5
H15B—C15A—H15C	109.5	H15E—C15—H15F	109.5
N2A—C16A—C17A	110.7 (4)	N1—C16—C17	110.7 (4)
N2A—C16A—H16A	109.5	N1—C16—H16C	109.5
C17A—C16A—H16A	109.5	C17—C16—H16C	109.5
N2A—C16A—H16B	109.5	N1—C16—H16D	109.5
C17A—C16A—H16B	109.5	C17—C16—H16D	109.5
H16A—C16A—H16B	108.1	H16C—C16—H16D	108.1
N1A—C17A—C16A	110.1 (4)	N2—C17—C16	109.9 (4)
N1A—C17A—H17A	109.6	N2—C17—H17C	109.7
C16A—C17A—H17A	109.6	C16—C17—H17C	109.7
N1A—C17A—H17B	109.6	N2—C17—H17D	109.7
C16A—C17A—H17B	109.6	C16—C17—H17D	109.7
H17A—C17A—H17B	108.2	H17C—C17—H17D	108.2
N1A—C18A—C19A	110.6 (4)	N2—C18—C19	110.1 (4)
N1A—C18A—H18A	109.5	N2—C18—H18C	109.6
C19A—C18A—H18A	109.5	C19—C18—H18C	109.6
N1A—C18A—H18B	109.5	N2—C18—H18D	109.6
C19A—C18A—H18B	109.5	C19—C18—H18D	109.6
H18A—C18A—H18B	108.1	H18C—C18—H18D	108.2
N2A—C19A—C18A	109.9 (4)	N1—C19—C18	111.0 (4)
N2A—C19A—H19A	109.7	N1—C19—H19C	109.4
C18A—C19A—H19A	109.7	C18—C19—H19C	109.4
N2A—C19A—H19B	109.7	N1—C19—H19D	109.4
C18A—C19A—H19B	109.7	C18—C19—H19D	109.4
H19A—C19A—H19B	108.2	H19C—C19—H19D	108.0
C21A—C20A—C25A	116.1 (4)	C25—C20—C21	116.9 (4)
C21A—C20A—N1A	124.3 (4)	C25—C20—N2	120.2 (4)
C25A—C20A—N1A	119.5 (4)	C21—C20—N2	122.8 (4)
C20A—C21A—C22A	121.4 (5)	C20—C21—C22	120.9 (5)
C20A—C21A—H21A	119.3	C20—C21—H21	119.5
C22A—C21A—H21A	119.3	C22—C21—H21	119.5
C23A—C22A—C21A	121.1 (5)	C23—C22—C21	120.1 (5)
C23A—C22A—H22A	119.4	C23—C22—H22	120.0
C21A—C22A—H22A	119.4	C21—C22—H22	120.0
C22A—C23A—C24A	119.0 (4)	C22—C23—C24	120.1 (4)
C22A—C23A—H23A	120.5	C22—C23—H23	119.9
C24A—C23A—H23A	120.5	C24—C23—H23	119.9
C25A—C24A—C23A	119.1 (5)	C23—C24—C25	118.8 (5)
C25A—C24A—H24A	120.5	C23—C24—H24	120.6
C23A—C24A—H24A	120.5	C25—C24—H24	120.6
F2—C25A—C24A	117.7 (5)	F1—C25—C20	119.5 (4)
F2—C25A—C20A	119.0 (4)	F1—C25—C24	117.4 (5)
C24A—C25A—C20A	123.2 (5)	C20—C25—C24	123.1 (5)
C8A—O1A—H1A1	109.5	C8—O1—H1B	109.5
C12A—O2A—C1A	110.9 (3)	C12—O2—C1	110.9 (3)
C2A—O4A—C3A	61.0 (3)	C2—O4—C3	61.3 (3)
C20A—N1A—C17A	115.4 (4)	C19—N1—C16	107.3 (3)
C20A—N1A—C18A	114.6 (4)	C19—N1—C13	111.3 (4)
C17A—N1A—C18A	110.2 (3)	C16—N1—C13	109.8 (4)
C19A—N2A—C13A	112.4 (4)	C20—N2—C18	115.7 (4)
C19A—N2A—C16A	107.7 (3)	C20—N2—C17	114.3 (4)
C13A—N2A—C16A	110.0 (4)	C18—N2—C17	111.4 (3)

### Hydrogen-bond geometry (Å, º)

**Table:**

*D*—H···*A*	*D*—H	H···*A*	*D*···*A*	*D*—H···*A*
O1—H1*B*···N1	0.82	2.19	2.979 (6)	163
O1*A*—H1*A*1···N2*A*	0.82	2.08	2.882 (6)	164
C1—H1···O3^i^	0.98	2.47	3.035 (7)	116
C13—H131···O3^ii^	0.97	2.51	3.394 (7)	152
C10—H10···O1	0.98	2.36	2.861 (6)	111
C10*A*—H10*A*···O1*A*	0.98	2.36	2.860 (6)	111
C13*A*—H13*A*···O3*A*^iii^	0.97	2.57	3.391 (6)	143
C11*A*—H11*A*···O3*A*^iv^	0.98	2.60	3.338 (6)	132
C15—H15*F*···O1^v^	0.96	2.42	3.375 (7)	171

Symmetry codes: (i) −*x*+1, *y*+1/2, −*z*+1; (ii) −*x*+1, *y*−1/2, −*z*+1; (iii) −*x*+2, *y*−1/2, −*z*; (iv) −*x*+2, *y*+1/2, −*z*; (v) −*x*+2, *y*+1/2, −*z*+1.
